# Let Food Be Thy Medicine: The Case of The Mediterranean Diet in Rheumatoid Arthritis

**DOI:** 10.31138/mjr.31.3.325

**Published:** 2020-09-30

**Authors:** Paraskevi Vranou, Athanasios Gkoutzourelas, Dorothea Athanatou, Efterpi Zafiriou, Maria G. Grammatikopoulou, Dimitrios P. Bogdanos

**Affiliations:** 1 Department of Rheumatology and Clinical Immunology, Faculty of Medicine, School of Health Sciences, University of Thessaly, Larissa, Greece; 2 Department of Dermatology, Faculty of Medicine, School of Health Sciences, University of Thessaly, Larissa, Greece; 3 Department of Nutritional Sciences and Dietetics, Faculty of Health Sciences, International Hellenic University, Alexander Campus, Thessaloniki, Greece

**Keywords:** NSAID, rheumatoid disease, DHA, EPA, docosahexaenoic acid, eicosatetraenoic acid, DAS28, dietary supplements, fish oil, omega-3, marine oil, anti-inflammatory nutrition

## Abstract

The role of diet in rheumatoid arthritis (RA) has been the topic of extensive research. The present review aimed to present and appraise the studies assessing adherence to the Mediterranean diet (MD) and the primary/secondary prevention of rheumatoid arthritis. Based on the available studies, the evidence appears low and adherence to the MD does not appear to affect RA indices.

## INTRODUCTION

Around 400 BC, Hippocrates was the first to realize the importance of food and diet in the primary and secondary prevention of disease.^1^ His moto, ‘let thy food be thy medicine and thy medicine be thy food’ has been followed for many centuries, and proved that dietary manipulations are effective in the management of several diseases and conditions.^2–5^

For rheumatoid arthritis (RA), an autoimmune rheumatic disease (ARD), in particular, to date, several researchers have addressed the effect of diet on RA indices. Among the examined dietary patterns, adherence to the Mediterranean diet (MD) appears to gain more residence, due to the fact that it combines a traditional dietary pattern, along with a life-style therapeutic remedy.^6,7^ In the MD, the distribution of macronutrients is 50% carbohydrates, 30% fats, and 20% proteins (60% of animal origin and 40% from vegetables). Total consumption of fat may be also around 40%, of which 20% are from monounsaturated fatty acids (MUFA). Thus, MD is mainly based on increased consumption of olive oil, whole grain, fruits, legumes, root vegetables, seeds and nuts, while dairy products, poultry and fish are less consumed. Red meat and eggs are less consumed as well. Wine is also consumed in low-to-moderate amounts (in non-Islamic countries). Salt intake is also limited.

The present review discusses the major findings of the clinical studies conducted in patients suffering from RA.

## ADHERENCE TO THE MD AND RISK FOR DEVELOPING RA

Using data from the Nurses’ Health Study (NHS, 1980–2008), Hu and associates^8^ performed a prognostic study in participants working in the Boston Women’s Hospital and Brigham in the United States. The study involved a total of 83,245 women nurses from the NHS, aged between 30 and 55 years and 91,393 women from the NHS II parallel study (1991–2009), aged 25–42 years old. The aim was to examine the relationship between adherence to the MD and the risk of developing RA in women in the United States. Participants at the time of the study were not affected by any connective tissue disease. To obtain nutritional information, subjects completed food frequency questionnaires (FFQs) every 4 years. The MD was assessed using the Alternative Mediterranean Diet Index (aMed),^9^ calculated based on the frequency of consumption of 9 food groups: whole grains, legumes, fruits, vegetables, fish, red meat, alcohol and monounsaturated-saturated fatty acids. During the follow-up years (28 years for the NHS and 20 years for the NHS II), 916 cases of RA were confirmed (631 cases in the NHS and 282 in the NHS II). In this study,^8^ adherence to the MD failed to reduce the risk for developing RA in either groups.

Between 1991 and 2011, on the premises of the Vasterbotten Intervention Program (VIP), Sundström and colleagues^10^ conducted a study in Sweden, investigating the effect of alcohol and diet on the development of RA. A total of 2,272 people participated and the mean time of evaluation before symptoms appeared was 7.7 years. Participants’ adherence to the Mediterranean diet was assessed using the Mediterranean Diet Score (MDS),^11^ calculated based on a FFQ of approximately 65 questions. Of the 2,272 people who took part in the VIP study, 386 developed RA and were identified by the medical records of the Rheumatology Department of Umea University Hospital. The remaining 1,886 formed the control group. Individuals who entered tertiary education showed a reduced risk of developing RA, while smokers showed an increased risk of RA. Of the 386 patients, the remaining 1,886 in the control group, 30 and 167, respectively, consumed four or more glasses of alcohol per week. Compared with those who consumed less than 1 glass per week, no correlation was observed with the occurrence of RA. No such correlation was also observed based on sex or type of alcohol (beer, wine, and other alcoholic beverages) or the presence of positive RF or anti-CCP. Evaluation of dietary pattern showed that Mediterranean diet (MD) in this study was not correlated with risk of developing RA with, some correlations in macronutrients such as: restriction of carbohydrate consumption among anti-CCP positive and protein consumption by smokers were reported; however, these correlations were not statistically significant. No association was observed between alcohol consumption or diet and the risk for developing RA. Additionally, alcohol consumption failed to demonstrate any differences in the incidence of RA, as proposed by other studies.

## CASE-CONTROL STUDIES ASSESSING MD ADHERENCE AMONG PATIENTS WITH RA

In 1999, Linos and associates^12^ conducted the first case-control study in Athens, Greece, assessing the consumption of olive oil, fish, vegetables, dietary constituents of the traditional Christian Orthodox diet/fasting and the risk for developing RA. The study, lasting for two years in total, included 145 patients with RA (83% females) and 188 subjects as a control group (78% females). Among the patients suffering from RA, 75% were positive for the rheumatoid factor (RF), while the remaining 25% were seronegative RA (RF negative). Also, 61% of the patients had bone erosions and 8% of them had subcutaneous nodules. According to Greek Orthodox fasting, the permitted days of consumption of olive oil are about 180 per year. The volunteers were divided into categories according to the fasting days per year. The risk of developing RA was significantly reduced in subjects with increased long-term consumption of olive oil, as well as in those with higher olive oil consumption compared to the lower consumption group. Average consumption of cooked vegetables ranged from 0.85 servings in the low quartile, to 2.9 servings in the fourth, upper quartile. The occurrence of RA was limited among those consuming increased amounts of cooked vegetables (OR = 0.39, 95% CI = 0.20, 0.77 and OR: 0.48, 95% CI: 0.25, 0.92 for the first and second highest consumption group, respectively). Based on this study, consumption of cooked vegetables and olive oil seems to have been an independent factor in reducing the incidence of RA. No other food category seemed to help limit the incidence of the disease. People who consumed small quantities of olive oil had a 2.5-fold higher risk of developing the disease.^12^

## EVIDENCE FROM RANDOMISED CONTROLLED TRIALS

In 2003, Sköldstam et al.^13^ conducted a randomised controlled study (RCT) in which the traditional Cretan MD was compared against the conventional Western-type diet (WD), in order to investigate the effect of diet on disease progression. The duration of the study was 3 months and included 51 patients with active RA, with at least 2 years of diagnosis, on stable treatment. Specifically, patients were divided into two separate groups: 26 patients in the intervention group (MD group) and 25 patients in the control group (WD group). All participants were fed for 3 weeks at the clinic’s restaurant (lunch and dinner), in order to improve patients’ compliance, according to the MD or WD diet standards, respectively. In the end of the trial, the intervention group demonstrated a significant decrease in disease activity score (DAS28) and in the overall health perception, investigated with the health assessment questionnaire (HAQ). Also, improvements were noted in the quality of life of participants, using the Short Form-36 (SF-36) questionnaire, while intake of non-steroidal anti-inflammatory drugs (NSAIDs) was not affected. The difference between the two groups was evident after the quarterly dietary intervention of MD, where its efficacy was detectable and statistically significant. Thus, the adoption of a MD model reduced the agility of the disease, increased functionality and improved the quality of life of patients.

In Sweden, Hagfors and associates^14^ conducted a 3-month nutritional intervention study, investigating whether a modified Cretan MD is able to alleviate RA signs and symptoms. The study included a total of 51 patients with well-controlled, active RA and disease duration of at least 2 years. An additional aim of this study was to determine the ability of patients to adapt to the experimental and controlled diets applied in the study, as well as to determine a method for better dietary history and nutritional status assessment of patients with RA. The group that followed the Cretan MD (intervention group, n = 26) showed a significant improvement in the disease activity index compared to the control group, which followed the conventional diet (control group, n = 25). The progress observed in the intervention group included a decrease in DAS28 and an improvement in physical - vital function. Additionally, 15 patients in the intervention group showed moderate or good clinical improvement due to change in disease activity, compared to 6 individuals from the control group. Patients following the MD pattern exhibited an increased antioxidant intake, including vitamin E, C and selenium intake.

Another intervention study, conducted by McKellar,^15^ aimed in promoting a Mediterranean-type diet among patients with RA, inhabiting socially deprived areas of Glasgow. The study included 130 adult women with RA, with an 8-year disease duration. Cooking lessons were provided to all patients in the intervention group (n = 75), with a particular emphasis on the MD pattern and relevant leaflets. The control group (n = 55) were only given written information on the MD. Significant improvements were observed in the intervention group compared to the controls, at 6 months post-baseline, concerning the disease’s clinical features, pain, morning stiffness and health assessment perception. Analysis of the food-frequency questionnaires revealed a significant increase in the weekly consumption of fruits, vegetables and legumes at 3 months. At the same time, an improvement was noted in the ratio of monounsaturated (MUFA) to saturated fat (SFA) intake, systolic blood pressure and weight loss, equal to 0.9 kg, on average. Participants appreciated the fact that they had participated in nutrition lessons. In conclusion, adherence to a MD pattern for 6 weeks was effective in increasing the consumption of “nutritious” foods. According to the authors,^15^ if the intervention was continued for a longer period of time, the MD could have served as a complementary therapy to treat RA.

Matsumoto^16^ conducted the prospective TOMORROW study to identify key elements of Mediterranean diet that make it possible to suppress RA indicators. The researchers concluded that the abundance in MUFA, which are a major component of the Mediterranean diet, appear to contribute to the suppression of the disease. Their study involved 208 patients with RA (intervention group) and 205 healthy volunteers (control group), of the same age and gender as participants in the prospective study, which is ongoing since the year 2010. Disease activity was assessed using the DAS28 questionnaire and erythrocyte sedimentation rate (ESR). The intake of MUFA in the intervention group was significantly lower than in the controls, whereas the ratio of MUFA/SFA appeared different. In addition, after adjustment for age, the DAS28 and ESR scores were significantly correlated with the MUFA/SFA intake os participants. Linear regression analysis revealed that high MUFA uptake was an independent predictor of RA remission. Changes in the DAS28 and ESR between 2010 and 2011 were associated with the MUFA/SFA intake ratio after adjustment for age. Based on the above, daily intake of MUFA, an essential component of the MD, may suppress disease activity in patients with RA.

## RESULTS FROM META-ANALYSES

Based on the above data, Forsyth and colleagues^17^ conducted a systematic review examining the role of the MD in the treatment of RA. In their review, four studies were analysed, already mentioned herein.^8,10,13,15^ According to the results, the evidence suggesting adherence to the MD for the prevention of RA is insufficient.

## WEIGHING THE EVIDENCE

Many studies have concluded that adherence to a healthy and balanced diet, rich in fish, olive oil and vegetables such as the MD, has a beneficial effect on the onset and progression of RA.^13,18^ Olive oil consists of the main fat component of MD. Oleic acid is the most widespread unsaturated fatty acid contained in olive oil (it reaches 75%), is converted to eicosatetraenoic acid and has anti-inflammatory properties similar to those found in omega-3 fatty acids found in fish oil.^13^ It has also been recognized as an antioxidant and anti-inflammatory food component. Hydroxytyrosol is another phenol contained in olive oil. The uptake of phenolic compounds increases LDL cholesterol and makes it stable against oxidation.^19,20^ Phenolic compounds reduce the inflammatory markers involved in atherosclerosis. Squalene, carotenoids, chlorophylls and α-tocopherol are some other antioxidants contained in olive oil.

Phytochemicals are chemicals found in plant foods (chlorophylls, carotenoids, flavonoids). Recent publications report that these chemicals play an important role in reducing chronic diseases such as: diabetes, atherosclerosis, asthma, RA, Alzheimer’s and cardiovascular disease. Eating vegetable foods on a daily basis, as defined in the MD, seems to reduce oxidative stress and inflammation.^21,22^

**Table 1.** Synopsis of the mean features of clinical studies which assessed the effect of Mediterranean diet in rheumatoid arthritis.

**First author**	**Country, Year**	**Type of study**	**RA patients involved (n)**	**Main features**	**Comments**
Skoldstam^13^	Sweden, 2003	Clinical trial	51	↓ DAS 28 ↓ HAQ ↓ Vital function ↓ SF-36	The clinical improvement was evident after 3 months of dietary intervention.
Hagfors^14^	Sweden, 2005	Clinical trial	51	↓ DAS28 ↓ physical-vital function ↓ antioxidants, vitamin E, C and selenium	The clinical improvement was evident after a 3-month nutritional intervention. The evaluation of dietary intake was quite accurate in identifying the foods consumed.
McKellar^15^	Glasgow, 2007	Clinical trial	130	↓ DAS 28 ↓ HAQ ↓ Morning stiffness	Dietary intervention was accepted by the participants. Adopting a Mediterranean diet for 6 weeks has beneficial effects.
Hu^8^	USA, 2015	Prospective study	916		No correlation was observed between the adoption of MD and the risk of developing RA.
Matsumoto^16^	Japan, 2017	Prospective study	208	↓ DAS 28 ↓ ESR	Daily consumption of MUFA, abundant in the MD, may limit the incidence of RA.
Sundström^10^	Sweden, 2014	Prospective study	386		No correlation was observed between alcohol consumption and diet on RA occurrence.
Linos^23^	Greece, 1999	Prospective study	145		Adherence to the Greek Orthodox fasting combined with olive oil consumption reduces the risk of RA.

DAS28: disease activity score, ESR: erythrocyte sedimentation rate, HAQ: Health Assessment Questionnaire, MD: Mediterranean Diet, MUFA: monounsaturated fatty acids, RA: Rheumatoid Arthritis

Finally, most of the ingredients of MD are characterized as functional foods with beneficial health effects. This was another reason why many scientists have conducted numerous studies analysing more and more nutrients and non-nutrients of this diet.

## CONCLUSION

In conclusion, circumstantial evidence appears to highlight the beneficial effect of MD in the development and treatment of RA. However, the clinical studies are few, and more work is needed at the clinical level with well-conducted clinical trials to reach safe conclusions regarding its true impact.
